# Population-level effect of HSV-2 therapy on the incidence of HIV in sub-Saharan Africa

**DOI:** 10.1136/sti.2008.029918

**Published:** 2008-09-16

**Authors:** R G White, E E Freeman, K K Orroth, R Bakker, H A Weiss, N O’Farrell, A Buvé, R J Hayes, J R Glynn

**Affiliations:** 1 London School of Hygiene and Tropical Medicine, London, UK; 2 Erasmus MC, University Medical Centre Rotterdam, Rotterdam, The Netherlands; 3 Pasteur Suite, Ealing Hospital, London, UK; 4 Institute of Tropical Medicine, Antwerp, Belgium

## Abstract

**Background:**

Herpes simplex virus type 2 (HSV-2) infection increases acquisition and transmission of HIV, but the results of trials measuring the impact of HSV-2 therapy on HIV genital shedding and HIV acquisition are mixed, and the potential impact of HSV-2 therapy on the incidence of HIV at the population level is unknown.

**Methods:**

The effects of episodic and suppressive HSV-2 therapy were simulated using the individual-level model *STDSIM* fitted to data from Cotonou, Benin (relatively low HIV prevalence) and Kisumu, Kenya (high HIV prevalence). Clinician- and patient-initiated episodic therapy, started when symptomatic, were assumed to reduce ulcer duration. Suppressive therapy, given regardless of symptoms, was also assumed to reduce ulcer frequency and HSV-2 infectiousness.

**Results:**

Clinician-initiated episodic therapy in the general population had almost no effect on the incidence of HIV. The impact of patient-initiated therapy was higher because of earlier treatment initiation, but still low (<5%) unless symptom recognition and treatment-seeking behaviour were very high. Suppressive therapy given to female sex workers (FSW) in Kisumu had little effect on population HIV incidence. In Cotonou, suppressive therapy in FSW with high coverage and long duration reduced population HIV incidence by >20% in the long term. Impact was increased in both cities by also treating a proportion of their clients. Long-term suppressive therapy with high coverage in the general population could reduce HIV incidence by more than 30%.

**Conclusions:**

These results show that HSV-2 therapy could potentially have a population-level impact on the incidence of HIV, especially in more concentrated epidemics. However, a substantial impact requires high coverage and long duration therapy, or very high symptom recognition and treatment-seeking behaviour.

HIV and herpes simplex virus type 2 (HSV-2) have a synergistic relationship. HIV affects HSV-2 shedding, ulcer recurrence rate and ulcer duration.^1^^–^5 HSV-2, in turn, has a strong impact on HIV transmission and acquisition and probably also affects the natural history of HIV infection.^2^ ^4^ ^6^^–^14 A meta-analysis of longitudinal studies found HSV-2 seropositivity to be associated with a risk ratio of HIV acquisition of 2.7 (95% confidence interval (CI) 1.9 to 3.9) in men and 3.1(95% CI 1.7 to 5.6) in women,^15^ and most cross-sectional studies have found a correlation between HSV-2 and HIV viral shedding and/or quantity.^10^ ^16^ ^17^

Antiviral therapy against HSV-2 could thus have a population-level impact on the global HIV epidemic in areas with a high HSV-2 prevalence such as sub-Saharan Africa.^4^ ^15^ ^18^ Two types of therapy exist: episodic and suppressive.

Episodic therapy is given to individuals with HSV-2 ulcers who notice their symptoms. Clinician-initiated therapy requires the patient to seek treatment for each ulcer episode, while patient-initiated therapy requires patients to self-medicate, reducing treatment delays. Three trials have assessed the effect of clinician-initiated episodic HSV-2 therapy on HIV infectiousness and results are currently available for two of these. A trial in women in Ghana and the Central African Republic found no significant impact on genital HIV RNA or time to ulcer healing.^19^ A trial in men in South Africa found a significant reduction in ulcer healing times and a borderline significant reduction in detection and quantity of ulcer HIV-1 shedding at day 7.^20^

Suppressive therapy is given to HSV-2 positive individuals regardless of whether they have symptoms. Six published trials have assessed the effect of HSV-2 suppressive therapy on genital HIV viral load in HIV/HSV-2-infected individuals who had not received antiretroviral therapy.^21^^–^26 Five found a significant reduction in frequency of genital shedding of HIV RNA and four found a significant reduction in the quantity of genital shedding of HIV RNA. All four studies that used valacyclovir or 800 mg twice daily acyclovir found a significant impact on frequency and quantity,^21^ ^23^ ^24^ ^26^ whereas only one of those using acyclovir 400 mg twice daily found an impact on frequency and neither had an impact on quantity.^22^ ^25^ The half-life of valacyclovir is about double that of acyclovir.^27^ Two trials have assessed the effect of acyclovir 400 mg twice daily on HIV acquisition.^28^ ^29^ No impact was observed in either trial,^28^ ^29^ suggesting that this dose may be too low for HIV prevention. The results of a randomised trial of suppressive therapy with acyclovir 400 mg twice daily on HIV transmission among discordant couples are expected in 2009.

Mathematical modelling can be used to explore the potential population-level impact of data from individual-level trials. Very few modelling studies have examined the relationship between HSV-2 and HIV,^30^^–^33 and none have examined the impact of HSV-2 antiviral therapy on the incidence of HIV in sub-Saharan Africa where such interventions are likely to prove most useful.

The aim of this paper is to explore the potential population-level impact of episodic and suppressive HSV-2 therapy on the incidence of HIV in one low and one high HIV prevalence city in sub-Saharan Africa.

## METHODS

### Data, model and baseline model scenarios

The mathematical model *STDSIM* has been fitted to data collected from four cities in sub-Saharan Africa as detailed in our previous publications.^33^ ^34^ The model was fitted to data from the Study Group on Heterogeneity of HIV epidemics in African Cities^35^ for the year 1997 and to available data on trends over time.

*STDSIM* is an individual-level stochastic model that simulates the natural history and interactions between HIV, HSV-2, syphilis, gonorrhoea, chlamydia and chancroid. It has been described in detail elsewhere.^32^ ^36^^–^39 *STDSIM* is able to simulate realistic sexual networks and heterogeneity between individuals in sexual behaviour and in the natural history of infection. It has also previously been used to explore the impact of treatment for sexually transmitted infections and male circumcision on HIV-1 prevention and the heterogeneous spread of HIV-1 in Africa, and the diverging HIV-1 and HIV-2 prevalence trends in West Africa.^33^ ^34^ ^40^ ^45^

The model representation of the natural history of sexually transmitted infections and, importantly, the interaction between HIV and other sexually transmitted infections were parameterised based on the literature where possible and poorly known parameter values have been subject to sensitivity analysis.^33^ ^34^ Full details are shown in Section S1 of the online supplement.

In this paper we present results for the two cities in the study with the lowest (Cotonou, Benin) and highest (Kisumu, Kenya) HIV prevalence. Cotonou represents an HIV epidemic highly concentrated among female sex workers (FSW) and their clients, while Kisumu represents a more generalised HIV epidemic typical of Eastern and Southern Africa.^34^

### Simulated interventions

For episodic and suppressive therapy we proposed a low, medium and high value for each of the required model parameters. The medium values were combined in one scenario to give the most likely estimate of impact and the low and high parameter values were combined in two further scenarios to give a plausible range for the impact of the intervention.

#### Episodic therapy

Clinician-initiated episodic therapy for HSV-2 was targeted at a proportion of symptomatic individuals who were simulated to recognise their ulcer, seek treatment and benefit from reduced ulcer duration. Full details are shown in Section S2 of the online supplement.

Patient-initiated episodic therapy for recurrent HSV-2 ulcers was targeted at a proportion of symptomatic individuals who were simulated to recognise their symptoms, self-treat and benefit from reduced ulcer duration. In this scenario we simulated clinician-initiated episodic therapy for primary ulcers (full details in Section S2 of the online supplement). For recurrent ulcers we assumed the same proportion recognised their symptoms as for clinic-based therapy, but assumed a higher proportion of those with recurrent ulcers received treatment because of the less frequent need for clinic visits, and assumed a greater reduction in ulcer duration because of the earlier initiation of treatment. Full details are shown in Section S3 of the online supplement.

For both clinician- and patient-initiated episodic therapy, the target group was the general population and the simulated intervention was ongoing from 1 January 2008.

#### Suppressive therapy

Suppressive HSV-2 therapy was initiated in a proportion of HSV-2 infected individuals who were simulated to start therapy and stop after a finite period of time. Suppressive therapy was assumed to increase the interval between ulcers, reduce the duration of ulcers and reduce the infectiousness of HSV-2 (but had no direct effect on HIV). We proposed a low, medium and high value for each of these parameters. Full details are shown in Section S4 of the online supplement.

Suppressive therapy was targeted at a proportion of the simulated population in a risk group. Individuals within this target group who were HSV-2 infected on 1 January 2008 and individuals who became HSV-2 infected after this date were simulated to receive suppressive therapy for 2 years, 10 years or until death. We assumed that, once individuals stopped suppressive treatment, they did not restart. The simulated target groups and coverage were: (1) 25%, 50%, and 75% of FSW; (2) 50% of FSW and 10%, 30% and 50% of male clients; (3) 10%, 30% and 50% of the general population.

### Outcome for episodic and suppressive therapy

We calculated the percentage reduction in the mean annual HSV-2 and HIV incidence among subjects aged 15–49 years in the general population over 5 and 20 years in the intervention scenarios compared with the baseline scenarios.

### Sensitivity analysis

We assessed the robustness of our findings to key baseline and intervention parameter values known to affect impact. Full details are shown in Section S5 of the online supplement.

## RESULTS

### Baseline scenario

A good fit of the model simulations to data for demography, sexual behaviour and epidemiology of the two populations was achieved and has been presented elsewhere.^33^ ^34^

**Figure 1:**
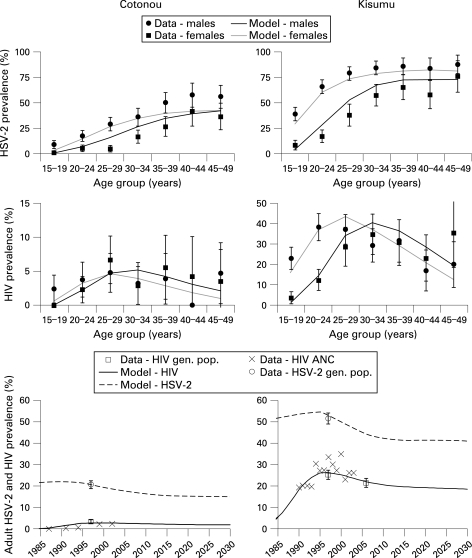
Observed (95% CI) and simulated prevalence of HIV and herpes simplex virus 2 (HSV-2) by age and sex in 1997 and over time in Cotonou and Kisumu (15–49 years). Note the difference in the y-axis scale used on HIV prevalence graphs. Gen pop, general population. ANC, antenatal clinic; F, female; M, male.

The fit of model-simulated HSV-2 prevalence to data from the two cities is shown in fig 1. The prevalence of HSV-2 in younger age groups was fitted preferentially because younger individuals account for a higher proportion of new HIV and HSV-2 infections. As a result, the prevalence of HSV-2 was underestimated in older women in Cotonou. In both sites the prevalence of HSV-2 in younger men was allowed to be overestimated in favour of fitting the prevalence of HSV-2 in younger women because higher-risk men may not have been completely captured in the original surveys.^46^ The fit of HSV-2 prevalence reflects the general age trends in HSV-2 prevalence and the important differences between the two sites, such as the difference in HSV-2 prevalence in women aged 15–19 years in Cotonou (9%) and Kisumu (39%).^47^ Owing to the sensitivity of the simulated prevalence of HIV and the prevalence of short-duration sexually transmitted infections to the change in sexual behaviour parameters, these parameters could not be further altered to improve the fit for HSV-2 without worsening the fit for HIV and the other sexually transmitted infections.

The model provided a reasonable fit to the age and sex patterns in HIV in both sites (fig 1). The model replicated the observed patterns, including the much higher prevalence of HIV among young women compared with young men. In Cotonou the prevalence of HIV was very similar among men and women, as observed. In Kisumu the prevalence of HIV peaked at a younger age in women than in men. The model fits the observed prevalence of HIV in the general population in 1997 and the available data on the trends in HIV prevalence reasonably well.

### Interventions

**Figure 2:**
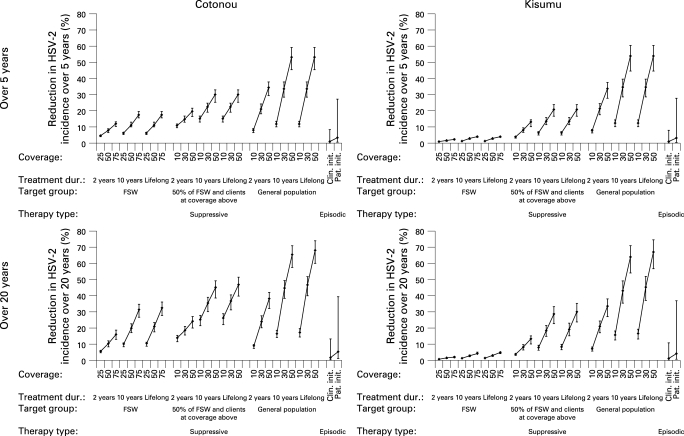
Impact of herpes simplex virus 2 (HSV-2) therapy on the incidence of HSV-2 over 5 and 20 years in adults aged 15–49 years. For each combination of therapy type, target group, treatment duration and coverage, three scenarios are shown corresponding to the “low” (⊥), “medium” (⧫) and “high” (⊤) parameter sets. See Methods for full details. FSW, female sex workers; Clin init, clinician initiated; Pat init, patient initiated.

**Figure 3:**
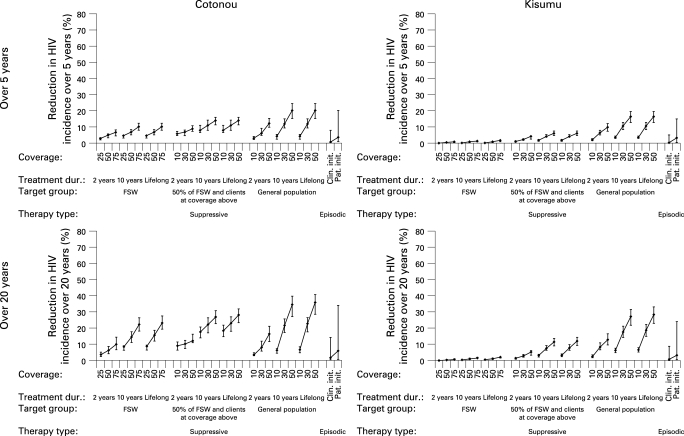
Impact of herpes simplex virus 2 (HSV-2) therapy on the incidence of HIV over 5 and 20 years in adults aged 15–49 years. For each combination of therapy type, target group, treatment duration and coverage, three scenarios are shown corresponding to the “low” (⊥), “medium” (⧫) and “high” (⊤) parameter sets. See Methods for full details. FSW, female sex workers; Clin init, clinician initiated; Pat init, patient initiated.

The effects of the interventions in terms of percentage reduction in HSV-2 and HIV incidence over 5 and 20 years in the two cities are shown in figs 2 and 3. The point estimates result from simulations assuming medium scenario options for all parameters, and the plausible bound from assuming all high or all low values. For simplicity, it is assumed that there are no other changes in interventions and no antiretroviral therapy over the period.

### Episodic therapy

With the medium scenario options, clinician-initiated episodic therapy had almost no effect on the incidence of HSV-2 or HIV in the short or long term in either city (right-hand side of graphs in figs 2 and 3). Patient-initiated therapy was only slightly more effective.

The upper plausible bound for episodic therapy, which assumes that the highest value of each parameter is correct, suggests that reductions in incidence are possible, especially with patient-initiated therapy. The sensitivity analysis of the effect of changing each parameter individually is shown in fig S1 in the online supplement. The increase in the proportion who recognise their symptoms had the largest effect on the impact on both HSV-2 and HIV incidence. Assuming 60% of individuals recognised their ulcers (and medium options for the other parameters), over 20 years clinician-initiated therapy reduced the incidence of HIV by 5.0% in Cotonou and 2.9% in Kisumu and patient-initiated therapy reduced the incidence of HIV by 15.1% and 9.8%, respectively.

### Suppressive therapy

Suppressive therapy in FSW in Kisumu had only a marginal effect on the incidence of HSV-2 and HIV, even with high coverage long-term therapy (figs 2 and 3). Treating half the FSW and a smaller proportion of their clients was more effective, leading to reductions in the incidence of HSV-2 of up to 30% and in the incidence of HIV of more than 10% in the long term.

In Cotonou, even giving long-duration suppressive therapy to FSW alone resulted in reductions of >30% in HSV-2 and >20% in HIV over the long term, and these were further increased by treating clients. Short-duration therapy (2 years) produced only small reductions in incidence in both cities.

Long-duration suppressive therapy in the general population can produce greater reductions in incidence over 20 years, with the effect rising steeply with the proportion of the population treated in both cities. With 30–50% of the population treated for 10 years, reductions in the incidence of HIV of 20–30% over 20 years were predicted (fig 3).

The sensitivity analysis of the effect of changing each intervention parameter value individually (see fig S2 in the online supplement) shows that the effect on HSV-2 infectivity has the largest effect on HSV-2 incidence, and that effects on ulcer duration are more important for HIV incidence. However, the results were fairly insensitive to altering any one parameter alone.

### Sensitivity analysis of baseline parameter values

Figure S3 in the online supplement shows the sensitivity of the results for episodic and suppressive therapy to the assumptions made about co-factor effects in different stages of HSV-2 infection. As expected, these changes had no impact on the incidence of HSV-2 incidence.

Assuming a between-ulcer co-factor effect, while keeping the population attributable fraction of HSV-2 on HIV transmission constant by reducing the co-factor effects during ulceration, reduced the impact of both episodic and suppressive therapy on the incidence of HIV in both cities. This is because therapy acts on the ulcer stages, so if they contribute less to the total co-factor effect, the potential benefit of therapy would be reduced.

Changing HSV-2 ulcer co-factors had larger impacts on the effects of treatment on the incidence of HIV, affecting both episodic and, particularly, suppressive therapy. However, co-factor effects of this magnitude do not fit well with the observed relative risks for the association of HSV-2 and HIV.

## DISCUSSION

This modelling study shows that effective treatment of HSV-2 could theoretically reduce the incidence of HIV sufficiently for a substantial public health impact. However, the impact depends on high coverage and long duration of therapy, or very high symptom recognition and treatment-seeking behaviour.

Episodic therapy would be easier to introduce and sustain than suppressive therapy as patients would already have presented for treatment and should be more motivated to continue. However, clinician-initiated therapy was shown to have very little population-level impact on the incidence of HIV. The impact of patient-initiated therapy was higher, but still low unless symptom recognition and treatment seeking was very high. Although studies have shown that individuals can be taught to recognise ulcers,^48^ this would be challenging to achieve on a large scale. The addition of acyclovir to syndromic management guidelines for countries with a high HSV-2 prevalence^49^ is only likely to have a population-level impact on HSV-2 and HIV if symptom recognition, treatment-seeking behaviour and correct syndromic management by providers can be substantially improved.

FSW might be easiest to target for suppressive therapy but, in the generalised HIV epidemic in Kisumu, suppressive therapy in FSW alone had almost no effect. There was an effect in the concentrated HIV epidemic in Cotonou, possibly because the proportion of HIV infections due to FSW and the population attributable fraction of HSV-2 on the incidence of HIV among FSW is higher in concentrated HIV epidemics. Additional treatment of clients increased the impact in both cities, suggesting that targeting core groups with high-risk behaviour, such as clients of FSW, can still be an effective strategy even in some generalised HIV epidemics, but only with high coverage and long duration. It is important to note that the impact of 10 years of suppressive therapy was almost as large as lifelong treatment. This suggests that HSV-2 treatment during the first few years following infection may avert most of the HIV infections preventable by HSV-2 therapy.

The general population scenarios show what could theoretically be achieved and underline the importance of HSV-2 in HIV transmission, but long-term treatment of large proportions of the population is unlikely to be feasible.

**Key messages**

- Herpes simplex virus type-2 (HSV-2) infection increases acquisition and transmission of HIV. Results from trials of HSV-2 therapy on HIV acquisition have been disappointing, perhaps because of insufficient herpes suppression. The potential impact of HSV-2 therapy on the incidence of HIV at the population level in sub-Saharan Africa is unknown.
- Simulated clinician-initiated episodic therapy in the general population had almost no effect on the incidence of HIV. The impact of patient-initiated therapy was higher because of earlier treatment initiation, but still low unless symptom recognition and treatment-seeking behaviour were very high.
- Simulated suppressive therapy in high-risk groups with high coverage and long duration in settings with concentrated epidemics reduced population HIV incidence in the long term. Long-term suppressive therapy with high coverage in the general population could substantially reduce the incidence of HIV.
- HSV-2 therapy could potentially have a population-level impact on HIV incidence, especially in more concentrated HIV epidemics. However, substantial impact requires high coverage and long duration therapy, or very high symptom recognition and treatment-seeking behaviour. To achieve the high coverage and long duration effects on HSV-2 that are required to have an important impact on the incidence of HIV, an effective vaccine against HSV-2 is needed.

The impact of HSV-2 therapy on the incidence and prevalence of HIV in the model is directly dependent on the assumed strength of the relationship between HSV-2 and HIV. The effects of treatment were dependent on assumptions about the level of the co-factor effect and the relative importance of co-factors between ulcers. Suppressive therapy might also reduce any between-ulcer co-factor effect, although this was not modelled, so the reductions in the effects of therapy in these alternative scenarios may have been overestimated.

Specific assumptions about the action and mechanism of the interventions on HSV-2 were informed by the literature wherever possible but, as many of these interventions are not yet well quantified or are hypothetical, a plausible range of values was explored for uncertain parameters. Even with these ranges, it is possible that our assumptions may have been over-optimistic in terms of some parameters such as the high parameter value for symptom recognition for episodic therapy. We assumed the same symptom recognition rate for both sexes, although it tends to be higher among men than women.50 We may therefore have underestimated the impact of episodic therapy in men and overestimated it in women. However, these effects will tend to cancel out in the results we presented on both sexes for episodic therapy.

We have presented the hypothetical reductions in HIV incidence and prevalence that could be achieved through HSV-2 interventions in certain scenarios. The real-world impact of scaled-up HSV-2 interventions would be tempered by logistical delivery constraints, such as the feasibility of funding and carrying out large-scale interventions in the general public, or of locating clients of FSW and persuading them of the utility of daily suppressive therapy for HSV-2, and by interactions with other HIV prevention and treatment programmes. Both episodic and suppressive therapies have limited potential as realistic HIV interventions. To achieve the high coverage and long duration effects on HSV-2 that are required to have an important impact on the incidence of HIV, an effective vaccine against HSV-2 is needed.
